# Mind the Gap: Model-Based Switching from Selatogrel to Maintenance Therapy with Oral P2Y12 Receptor Antagonists

**DOI:** 10.3390/biom13091365

**Published:** 2023-09-08

**Authors:** Chih-hsuan Hsin, Jasper Dingemanse, Andrea Henrich, Corine Bernaud, Martine Gehin, Andreas Krause

**Affiliations:** Department of Clinical Pharmacology, Idorsia Pharmaceuticals Ltd., 4123 Allschwil, Switzerland

**Keywords:** emergency treatment, injections, myocardial infarction, P2Y_12_ receptor antagonists, platelet aggregation

## Abstract

**Background:** The P2Y_12_ receptor antagonist selatogrel is being developed for subcutaneous self-administration with a ready-to-use autoinjector at the onset of acute myocardial infarction (AMI) symptoms. The unique pharmacological profile of selatogrel (fast, potent, and short-acting) can bridge the time gap between the onset of AMI and first medical care. A clinical Phase 1 study showed a time-dependent pharmacodynamic interaction between selatogrel and loading doses of clopidogrel and prasugrel. As treatment switching is a common clinical practice, the assessment of subsequent switching from a clopidogrel loading dose to the first maintenance dose of oral P2Y_12_ receptor antagonists is highly relevant. **Objectives:** Model-based predictions of inhibition of platelet aggregation (IPA) for the drugs triggering pharmacodynamic interactions were to be derived to support clinical guidance on the transition from selatogrel to oral P2Y_12_ receptor antagonists. **Methods:** Scenarios with selatogrel 16 mg administration or placebo followed by a clopidogrel loading dose and, in turn, prasugrel or ticagrelor maintenance doses at different times of administration were studied. Population pharmacokinetic/pharmacodynamic modeling and simulations of different treatment scenarios were used to derive quantitative estimates for IPA over time. **Results:** Following selatogrel/placebo and a clopidogrel loading dose, maintenance treatment with ticagrelor or a prasugrel loading dose followed by maintenance treatment quickly achieved sustained IPA levels above 80%. Prior to maintenance treatment, a short time span from 18 to 24 h was identified where IPA levels were predicted to be lower with selatogrel than with placebo if clopidogrel was administered 12 h after selatogrel or placebo. Predicted IPA levels reached with placebo alone and a clopidogrel loading dose at 4 h were consistently lower than with selatogrel administration, followed by a clopidogrel loading dose at 12 h. If a clopidogrel loading dose is administered at 12 h, selatogrel maintains higher IPA levels up to 16 h. IPA levels are subsequently lower than on the placebo until the administration of the first maintenance dose. **Conclusions:** Model-based predictions informed the transition from selatogrel subcutaneous administration to oral P2Y_12_ therapy. The application of modeling techniques illustrates the value of employing pharmacokinetic and pharmacodynamic modeling for the simulation of various clinical scenarios of switching therapies.

## 1. Introduction

### 1.1. P2Y_12_ Receptor Antagonists

P2Y_12_ receptors play a crucial role in platelet aggregation. Early administration of acetylsalicylic acid and a P2Y_12_ receptor antagonist, i.e., oral dual antiplatelet therapy, is part of the standard of care for acute coronary syndrome (ACS) management [1,2,3].

Approved oral P2Y_12_ receptor antagonists include clopidogrel, prasugrel, and ticagrelor [4,5,6]. Clopidogrel is still the most widely used P2Y_12_ receptor antagonist [7,8,9,10] with irreversible and competitive P2Y_12_ receptor binding [11]. Clopidogrel is a prodrug with a slow onset of action (2 to 6 h), limited platelet inhibition (20% to 50%), and high inter-individual pharmacodynamic (PD) variability [11,12,13]. Prasugrel competes with ADP for binding to the P2Y_12_ receptor, establishing an irreversible bond [14,15]. The inhibition of platelet aggregation (IPA) of prasugrel is faster (0.5 to 4 h) and stronger than with clopidogrel [15]. Ticagrelor is the first approved reversible oral P2Y_12_ receptor antagonist [16]. Ticagrelor and its active metabolite both bind non-competitively with ADP to the P2Y_12_ receptor [16,17]. The onset of the action of ticagrelor is similar to that of prasugrel, achieving >80% IPA between 0.5 and 4 h [16,18]. Compared with clopidogrel, prasugrel and ticagrelor have demonstrated better efficacy in reducing the incidence of cardiovascular events in patients with ACS [19,20].

The American College of Cardiology/American Heart Association (ACC/AHA), the European Society of Cardiology (ESC), and the Asian Pacific Society of Cardiology (APSC) recommend ticagrelor or prasugrel over clopidogrel for the treatment of patients with AMI [1,2,3]. Specifically, the 2020 APSC consensus recommendations highlight the higher prevalence of polymorphisms affecting CYP2C19 function in the Asian population and the consequent bioactivation and bioavailability of clopidogrel [3,21].

### 1.2. Selatogrel

Selatogrel is a novel potent, selective, reversible, and competitive P2Y_12_ receptor antagonist with a rapid onset of action in clinical development for the emergency treatment of AMI [22,23,24]. Selatogrel is administered subcutaneously (s.c.) and was shown to be safe and well tolerated in seven Phase 1 and two Phase 2 clinical studies. Median time to maximum plasma concentration was 0.5 to 0.75 h, distribution half-life was 1 to 2 h, and terminal half-life was 4 to 7 h [22,23,25,26,27,28,29,30,31]. Selatogrel is not metabolized by major CYP enzymes and is primarily eliminated unchanged via the biliary route [27]. Therefore, changes in CYP enzyme and transporter activities are unlikely to affect its pharmacokinetics (PK) and PD [27,28]. In vitro, selatogrel did not induce CYP enzymes or inhibit CYP enzymes and transporters; hence, PK drug-drug interactions (DDIs) with concomitant medications are very unlikely [27,28]. Selatogrel showed a rapid onset of action (within 15 min) and potent IPA (>85%), lasting 6 to 8 h with a return to baseline within 24 h at doses of 8 mg and 16 mg studied in Phase 2 [22,23].

Preclinical experiments demonstrated the potent anti-thrombotic properties of selatogrel by preventing and dissolving platelet thrombi without disrupting hemostatic seals, while the off-target activity of clopidogrel and ticagrelor destabilized mural platelet thrombi [32]. Subcutaneous application rapidly inhibited ongoing thrombosis in guinea pigs and mice, even normalizing blood flow in a thrombosis model [33].

### 1.3. Clinical Study Data

In a Phase 1 study, PD DDIs between selatogrel and prasugrel, as well as between selatogrel and clopidogrel, were observed [29]. No PD interaction was observed with ticagrelor following selatogrel administration since it binds reversibly to P2Y_12_ at a site distinct from the ADP binding site. The receptor remains functional, i.e., available for binding, after the dissociation of ticagrelor.

The data from the two Phase 2 studies in patients with chronic coronary syndrome and acute myocardial infarction suggested an additional reduction in platelet residual activity when selatogrel was given on top of any background oral P2Y_12_ receptor antagonist. The baseline or pre-dose level of platelet reactivity was recovered within 24 h of selatogrel administration [22,23].

### 1.4. Drug-Drug Interaction Modeling

Using the data of the PD DDI study, PK/PD modeling and simulation showed that administration of a prasugrel loading dose of 4.5 h after selatogrel resulted in a clinically negligible DDI with IPA remaining >80% at 24 h post-selatogrel dosing [34]. This level of API was predicted to be maintained subsequently by the administration of o.d. prasugrel maintenance doses.

As selatogrel and clopidogrel compete for the same P2Y_12_ receptor binding site, the PD DDI between selatogrel and clopidogrel was pronounced for several hours after administration of selatogrel [29,34] since receptors were occupied by selatogrel at the time of clopidogrel administration. Due to the short half-life (approximately 30 min) of the active metabolite of clopidogrel [4], most of them are eliminated before selatogrel is released from the P2Y_12_ receptors.

### 1.5. Research Question

All switch scenarios aim to investigate optimal timing to ensure the maintenance of sufficient IPA. The scenarios of switching from selatogrel to ticagrelor or prasugrel were not considered critical based on the clinical DDI study data. The focus of this work was therefore on the transition to a clopidogrel loading dose to prevent suboptimal IPA, as the PD interaction between selatogrel and clopidogrel was the most pronounced DDI.

The aim of this research was to investigate the switch from selatogrel to clopidogrel loading dose, followed by the three different loading or maintenance treatments (ticagrelor, prasugrel, or clopidogrel).

## 2. Materials and Methods

The PK and PD data from 4 Phase 1 studies (single-ascending doses [26], drug-drug interaction [29], mass balance and metabolism [27]) and 2 Phase 2 studies (selatogrel in patients with chronic coronary syndrome [23] and in patients with acute myocardial infarction [22]) were described well by a semi-mechanistic PK/PD model [34]. 

Population PK models are mathematical descriptions, i.e., differential equations, that are able to describe and predict drug flows in a body. The body is described by compartments: the drug depot (here, the subcutaneous space), the central compartment (the bloodstream), and the peripheral compartments (tissue).

With a specified amount of drug administered into the depot compartment (the subcutaneous space here), the mathematical equations mimic drug distribution to the other compartments with estimated transfer rates and clearance from the central compartment. The outcome is a prediction of drug concentration over time on a dense grid of time points. The model is validated against the data at the observation time points. 

Subsequently, drug concentration is linked to the effect of PD (here, platelet reaction units [PRU] and, in turn, IPA). The selatogrel model includes an effect compartment that describes the appearance and disappearance of free receptors. Drugs in the central compartment bind to the free receptors to yield receptor-drug complexes. As with PK, the fraction of free receptors is predicted on a fine-time grid. From this, PRU estimates are derived as a function of PRU at baseline and currently free receptors. The PD model was built on and validated against a dedicated DDI study [29]. All technical details are provided in [34].

A key advantage of PK/PD models of data is that the models are able to predict any dose and related effect at any given time point, in particular doses and time points not studied in the clinic.

**Figure A1:**
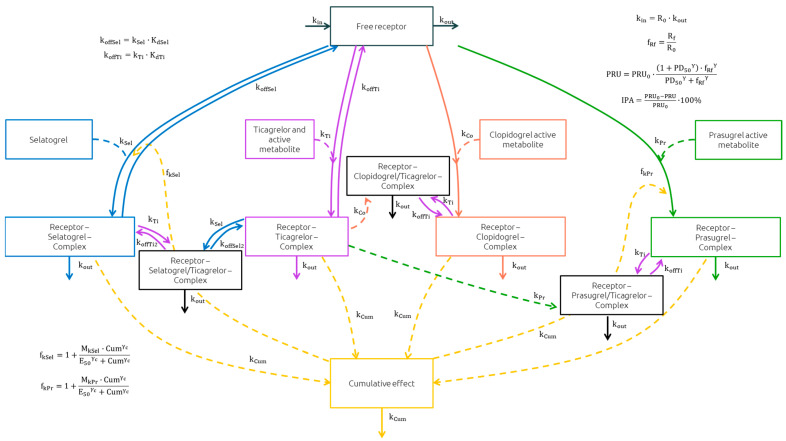
Selatogrel semi-mechanistic PD model. Cum, cumulative effect; E50, half-maximum for cumulative effect; fkPr, factor resulting from cumulative effect modulating prasugrel binding; fkSel, factor resulting from cumulative effect modulating selatogrel binding; fRf, fraction of free receptor; IPA, inhibition of platelet aggregation; kClo, clopidogrel binding rate constant to receptor; kCum, rate constant for cumulative effect; KdSel, selatogrel dissociation constant; KdTi, ticagrelor dissociation constant; kin, formation rate constant of receptor; koffSel, dissociation rate constant of receptor complex with selatogrel; koffSel2, dissociation rate constant of receptor complex with selatogrel and ticagrelor; koffTi, dissociation rate constant of receptor complex with ticagrelor; koffTi2, dissociation rate constant of receptor complex with selatogrel and ticagrelor; kout, elimination rate constant of receptor; kPr, prasugrel binding rate constant to receptor; kSel, selatogrel binding rate constant to receptor; kTi, ticagrelor allosteric binding rate constant to receptor; MkPr, prasugrel maximum cooperativity effect; MkSel, selatogrel maximum cooperativity effect; PD50, half-maximum for PRU signal; PRU, P2Y_12_ receptor units; PRU0, baseline P2Y_12_ receptor units; R0, baseline receptor; Rf, free receptor; γ, Hill coefficient. Modified from Henrich et al. [34].

To predict complex clinical situations with more than one P2Y_12_ receptor antagonist following selatogrel, the model was extended with a component for the interaction between competitive (clopidogrel and prasugrel) and non-competitive (ticagrelor) P2Y_12_ receptor antagonist binding (Figure A1) using a reference subject with a body weight of 70 kg and a PRU of 200 (naïve of treatment). 

Using the extended model, simulations were conducted to predict possible clinical scenarios. The scenarios simulated 16 mg selatogrel s.c. or placebo at time 0, followed by a loading dose of clopidogrel and subsequent loading and maintenance doses of ticagrelor or prasugrel.

**Figure 1:**
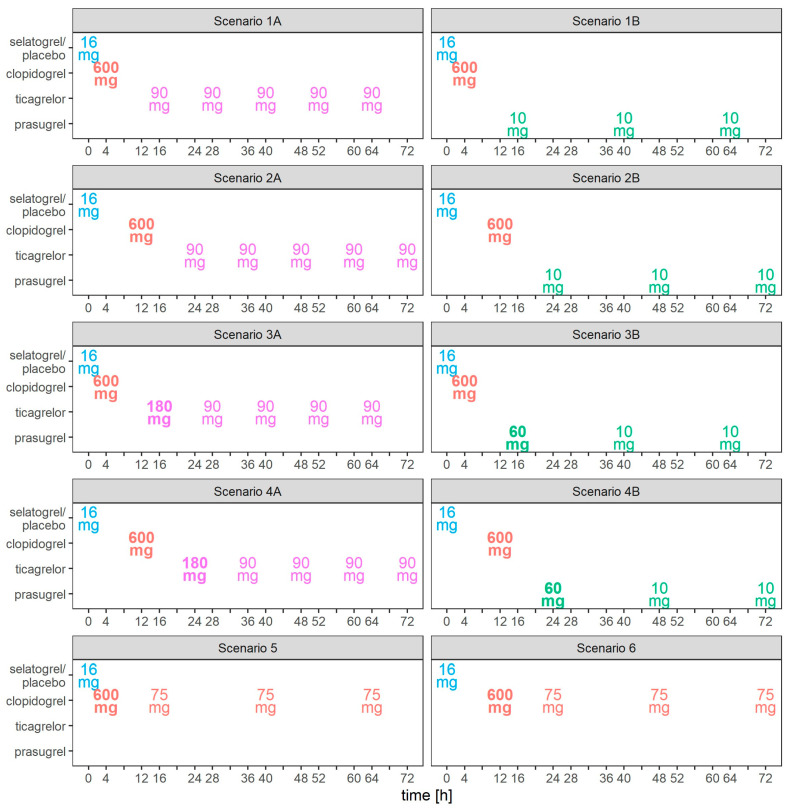
Scenarios. Colors indicate last treatment, selatogrel/placebo (blue), clopidogrel (red), ticagrelor (purple), or prasugrel (green). Font face indicates loading (boldface) or maintenance doses. The time axis displays only times of drug administration.

Ten scenarios were considered: in each scenario, 16 mg selatogrel or placebo was simulated as being administered s.c. at time 0 h (i.e., shortly after occurrence of AMI symptoms), followed by a 600 mg loading dose of clopidogrel at 4 or 12 h and subsequent administration of either ticagrelor, prasugrel, or clopidogrel (Table 1, Figure 1).

IPA was predicted every 0.1 h up to 72 h after selatogrel/placebo administration. A target PRU below 100 was defined as the PD target in previous Phase 2 studies [22,23], corresponding to IPA > 80% [35,36]. In all simulations, the maximum IPA and duration of IPA above 80% were derived after each simulated loading and maintenance dose of ticagrelor, prasugrel, and clopidogrel. 

The PK/PD model was based on a longitudinal mixed-effects model [34,37,38,39]. Simulations were performed with Simulx 2021R1 [40]. R version 4.0.4 [41] was employed for numerical result derivations and visualization of results.

## 3. Results

**Figure 2:**
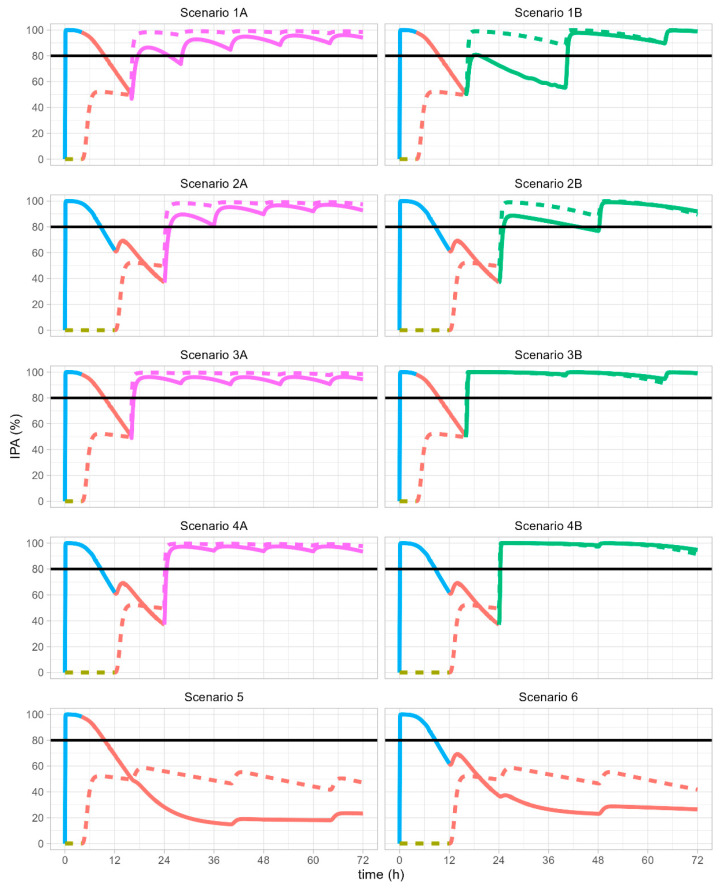
Inhibition of platelet aggregation vs. time in different treatment scenarios. IPA (%), inhibition of platelet aggregation in percent. Black lines indicate IPA of 80%. Solid lines indicate effects with initial selatogrel administration. Dashed lines indicate effects with initial placebo administration. Colors indicate the last treatment, selatogrel (blue), placebo (olive), clopidogrel (red), ticagrelor (purple), or prasugrel (green).

The scenarios where a placebo is administered instead of selatogrel aim to reflect the current standard of care for AMI patients receiving oral P2Y_12_ receptor antagonists. All scenarios were assessed with clopidogrel loading doses administered 4 h and 12 h after selatogrel. The following text generally refers to scenarios (Table 1, Figure 1) and results (Table 2, Figure 2) by color and line type. Figure numbers are not repeatedly referenced to facilitate reading.

### 3.1. Selatogrel/Placebo Followed by Clopidogrel Loading Dose Administration

A clopidogrel loading dose given 4 h after placebo was predicted to achieve approximately 50% IPA (scenarios 1A and 1B, olive and red dashed lines). The results are identical with an 8 h time shift if the clopidogrel loading dose is administered 12 h after the placebo (scenarios 2A and 2B).

If a clopidogrel loading dose is administered 4 h or 12 h after selatogrel, the predicted IPA levels are substantially higher than after placebo over 18 h (scenarios 1A, 1B, 2A, and 2B; Figure 2, blue and red solid lines), but lower than after placebo between 18 and 24 h if a clopidogrel loading dose is administered 12 h after selatogrel. 

If a clopidogrel loading dose is administered at 12 h and followed by ticagrelor or prasugrel administration at 24 h, predicted IPA levels between 18 h and 24 h are lower if selatogrel is the initial time 0 h treatment compared with the placebo, with minimum IPA of 37% and 49% (scenarios 2A, 2B, 4A, and 4B) for selatogrel and placebo, respectively.

**Figure 3:**
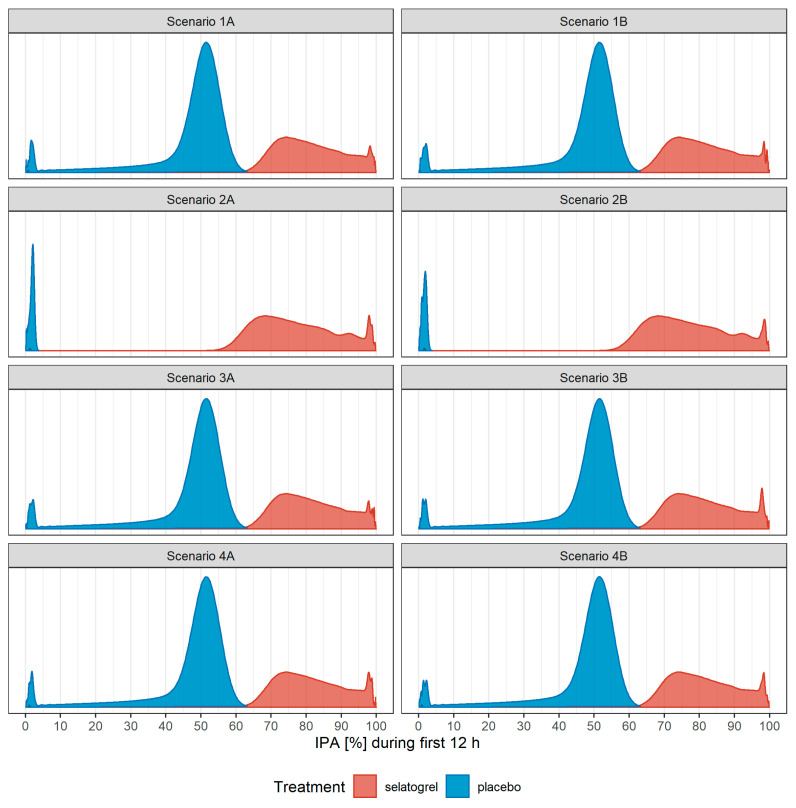
Distribution of predicted %IPA in the first 12 h with different treatment scenarios. Nonparametric density estimation of distributions of model-predicted %IPA for each scenario. IPA (%), inhibition of platelet aggregation in percent.

Overall, initial selatogrel administration yielded substantially higher IPA levels compared with the initial placebo administration over the first 12 h (Figure 3).

### 3.2. Ticagrelor Maintenance Therapy

**Figure A2:**
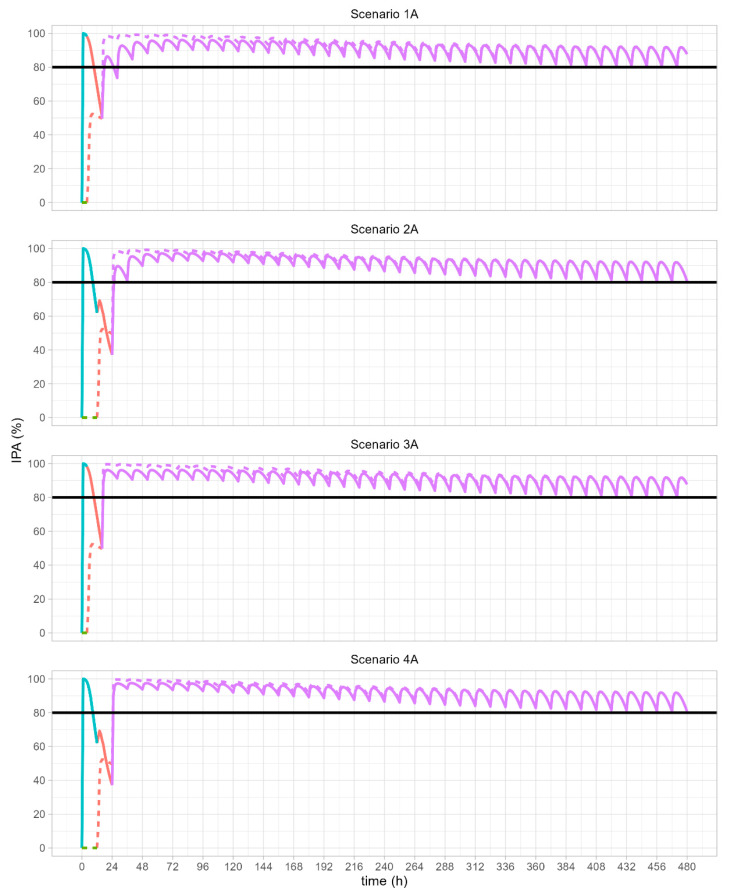
Inhibition of platelet aggregation vs. time over 20 days (different scenarios). IPA (%), inhibition of platelet aggregation (IPA) in percent. Black horizontal lines indicate IPA of 80%. Solid and dashed lines indicate effects with initial selatogrel and placebo administration, respectively. Colors indicate the last treatment, selatogrel (turquoise blue), placebo (olive), clopidogrel (red), or ticagrelor (purple). IPA levels decrease slowly with time due to the sustained effects of clopidogrel.

Following selatogrel/placebo and a clopidogrel loading dose at 4 h or 12 h, subsequent b.i.d. administration of a ticagrelor 90 mg maintenance dose maintains IPA levels above 90% from 25 h onwards on selatogrel and placebo (scenarios 1A and 2A, purple solid and dashed lines). A ticagrelor loading dose of 180 mg increases IPA further to 94% and 98% within an hour after ticagrelor dosing (scenarios 3A and 4A). Following initial administration of selatogrel or placebo, differences in IPA with repeated ticagrelor dosing disappear slowly but are not clinically relevant, with IPA levels consistently exceeding 80% (Figure A2), i.e., above what would be achieved with clopidogrel alone. 

### 3.3. Prasugrel Maintenance Therapy

After one dosing interval of prasugrel at the 10 mg maintenance dose following clopidogrel loading, the predicted IPA was 55% (with initial selatogrel) and 88% (with initial placebo) in scenario 1B and 77% and 88% in scenario 2B, respectively. Substantially higher predicted IPA levels were observed if a clopidogrel loading dose was followed by a 60 mg loading dose of prasugrel (scenarios 3B and 4B, green lines) and almost no difference between selatogrel and placebo, with all IPA levels predicted to exceed 97%.

### 3.4. Ticagrelor and Prasugrel Maintenance Therapy

If a clopidogrel loading dose was simulated to be given 4 h after selatogrel or placebo and followed by ticagrelor or prasugrel 12 h later, the predicted IPA levels were consistently above what would be achieved with administration of clopidogrel alone (placebo simulation, purple lines in scenarios 1A, 1B, 3A, and 3B). 

Administration of a ticagrelor loading dose (scenarios 3A and 4A) or maintenance dose (scenarios 1A and 2A) was predicted to rapidly achieve IPA >80%. In contrast, while a prasugrel loading dose (scenarios 3B and 4B) also quickly achieved IPA >80% over the entire dosing interval, prasugrel maintenance dosing required 24 h (a second maintenance dose) to exceed 80% IPA over the entire dosing interval (scenarios 1B and 2B).

With either selatogrel or placebo, a loading dose of ticagrelor or prasugrel was predicted to achieve 80% IPA over a dosing interval (scenarios 3A to 4B), while a similar PD effect with maintenance doses alone was achieved only after the second maintenance dose of ticagrelor or prasugrel (scenarios 1A to 2B).

### 3.5. Clopidogrel Maintenance Therapy

Following selatogrel/placebo and a clopidogrel loading dose, clopidogrel maintenance doses were predicted to show maximum IPA below 60% and as low as 20% between 24 h and 72 h following initial selatogrel administration (scenarios 5 and 6). 

## 4. Discussion

Delayed time to AMI treatment (i.e., time from AMI symptoms to first medical contact) is associated with increased mortality [42,43]. Treatment initiation delays can vary widely due to patient-delayed calls for emergency medical services as well as system delays, such as transfers to the hospital [44,45].

Selatogrel is the first P2Y_12_ receptor antagonist developed for s.c. self-administration using a pre-filled autoinjector at the onset of suspected AMI symptoms. Clinical studies in healthy subjects and Phase 2 studies provided extensive information on the PK and PD of selatogrel [22,23,25,26,27,28,29,30,31]. The ongoing global clinical Phase 3 study [46] evaluates the efficacy and safety of selatogrel in addition to the standard of care in patients with recent AMI. 

Although ticagrelor and prasugrel are recommended as first-line oral P2Y_12_ therapies in AMI [1,2,3], clopidogrel is still widely used [7,8,9,10] due to the availability of generic forms of clopidogrel but also increased bleeding risk with the newer, more potent P2Y_12_ receptor antagonists [7,8,44]. Given the limited PD effect achieved by clopidogrel, international expert consensus recommends that patients who have received a loading dose of clopidogrel receive another loading dose of ticagrelor or prasugrel before PCI to prevent stroke or thrombosis [47]. 

Model-based simulations were used to evaluate clinically relevant scenarios if a s.c. self-administration of selatogrel is given at the onset of suspected AMI symptoms prior to AMI standard-of-care treatment. The focus was on the transition from an initial clopidogrel loading dose given after selatogrel self-administration to clopidogrel maintenance dosing or switching from the clopidogrel loading dose to ticagrelor or prasugrel loading or maintenance dosing. 

The PK/PD simulations were based on models for healthy subjects. Two Phase 2 studies showed that the onset of action of selatogrel and maximum IPA achieved in chronic coronary syndrome and AMI patients are comparable to the effects in healthy subjects [22,23]. 

The simulations were in line with the clinical data, showing that administration of selatogrel achieves fast and sustainable IPA levels >80%. As selatogrel dissociates from P2Y_12_ receptors, IPA decreases while remaining consistently above the IPA level achieved with a loading dose of clopidogrel alone during the first 12 h. These predicted IPA levels are consistent with observations in healthy subjects up to 12 h after selatogrel administration followed by a loading dose of clopidogrel [29] and comparable to the peak effect of a loading dose of clopidogrel (31.8 ± 21.1%) in patients undergoing cardiac catheterization [15].

If a clopidogrel loading dose is administered 12 h after selatogrel, a small window between 18 and 24 h exists in which the combined PD effect is slightly lower than the effect of clopidogrel alone. Subsequent transition to clopidogrel maintenance dosing results in suboptimal IPA with IPA lower than with clopidogrel monotherapy. 

Switching from a clopidogrel loading dose to ticagrelor or prasugrel achieves potent and sustained IPA compared with clopidogrel-only treatment over 72 h. Ticagrelor and prasugrel both achieve higher IPA if administered 12 h after a clopidogrel loading dose followed by maintenance doses [48,49]. Clopidogrel maintenance doses, following a clopidogrel loading dose, however, do not achieve IPA above 60% and levels as low as 20% IPA after 24 to 72 h, such that clopidogrel maintenance therapy is not ideal.

Overall, the simulations indicated that administration of selatogrel followed by a clopidogrel loading dose and subsequently ticagrelor or prasugrel generally achieves higher IPA than clopidogrel-only treatment, reported to achieve approximately 50% IPA on maintenance dosing [12]. The interaction between selatogrel and clopidogrel causes IPA to be below clopidogrel-only treatment in a short time interval of 6 h, i.e., at 18 to 24 h after selatogrel, if a clopidogrel loading dose is administered 12 h after selatogrel (scenario 2A). This is not the case if the clopidogrel loading dose is administered earlier, i.e., 4 h after selatogrel (scenario 1A). 

In subjects without background P2Y_12_ receptor antagonist therapy in a Phase 1 clinical study, PD DDIs between selatogrel and clopidogrel, as well as between selatogrel and prasugrel, were observed. In patients with chronic coronary syndrome on background oral P2Y_12_ receptor antagonist therapy [23], a single injection of selatogrel achieved additional IPA for a few hours, with the extent depending on the background oral P2Y_12_ receptor antagonist therapy. These model-based predictions therefore apply only to patients who are not on background P2Y_12_ receptor antagonist therapy at the time of their recurrent AMI. 

The model-based approach has limitations. The model was developed based on rich PK and PD data of selatogrel and rich PD data of clopidogrel, prasugrel, and ticagrelor from clinical studies [22,23,26,27,28,29]. Individual PK data for clopidogrel, prasugrel, and ticagrelor were not available, so the PK models of clopidogrel, prasugrel, and ticagrelor were built from published models [45,48,50] with population-typical PK parameter estimates. Inter-individual variability in the combination of therapies could therefore not be assessed. This is particularly relevant for clopidogrel and its active metabolite, which is associated with large inter-individual variability in both PK and PD [50,51]. The Phase 1 study investigated PD DDIs between selatogrel and loading doses of each of the three oral P2Y_12_ receptor antagonists [29]. The prediction model includes some assumptions, e.g., that the change in binding kinetics of the competitive inhibition of ticagrelor and selatogrel without the two compounds bound to the receptor is proportional to the binding kinetics with the two compounds bound to the receptor. The model was shown to describe the available data well, supporting the validity of the assumptions.

In an ideal world, a study with all treatments (selatogrel, clopidogrel, prasugrel, and ticagrelor) and measurements of all drug concentrations and resulting IPA would allow for quantification of variability and, e.g., estimation of the percentage of patients predicted below thresholds such as 80% IPA.

This model-based approach is a major step forward and enables us to study such scenarios quantitatively in the absence of clinical data.

## 5. Conclusions

Model-based predictions can be useful tools for studying complex clinical scenarios and complement clinical evidence. The results underscored that high IPA levels are reached after subcutaneous administration of selatogrel, and these IPA levels are predicted to be maintained if a subsequent clopidogrel loading dose (600 mg) is followed by either ticagrelor or prasugrel rather than by a clopidogrel maintenance dose (75 mg).

The application of modeling techniques illustrates the value of employing PK/PD modeling for the simulation of various clinical scenarios of switching therapies, not limited to the application shown here.

## Figures and Tables

**Table 1:** Simulation scenarios. Boldface font indicates loading doses. Times are relative to selatogrel/placebo administration.

Scenario	P2Y_12_ Receptor Antagonist Administration Schedule			
	Selatogrel/Placebo	Clopidogrel	Ticagrelor	Prasugrel
*Scenarios without loading dose of ticagrelor/prasugrel*				
1A	16 mg at 0 h	600 mg at 4 h	90 mg at 16 h and every 12 h subsequently	
1B	16 mg at 0 h	600 mg at 4 h		10 mg at 16 h and every 24 h subsequently
2A	16 mg at 0 h	600 mg at 12 h	90 mg at 24 h and every 12 h subsequently	
2B	16 mg at 0 h	600 mg at 12 h		10 mg at 24 h and every 24 h subsequently
*Scenarios with loading dose of ticagrelor/prasugrel*				
3A	16 mg at 0 h	600 mg at 4 h	**180 mg** at 16 h and 90 mg every 12 h subsequently	
3B	16 mg at 0 h	600 mg at 4 h		**60 mg** at 16 h and 10 mg every 24 h subsequently
4A	16 mg at 0 h	600 mg at 12 h	**180 mg** at 24 h and 90 mg every 12 h subsequently	
4B	16 mg at 0 h	600 mg at 12 h		**60 mg** at 24 h and 10 mg every 24 h subsequently
*Scenarios with clopidogrel only*				
5	16 mg at 0 h	600 mg at 4 h 75 mg at 16 h and every 24 h subsequently		
6	16 mg at 0 h	600 mg at 12 h 75 mg at 24 h and every 24 h subsequently		

**Table 2:** Predicted pharmacodynamic effects during the maintenance period with different treatment combinations. IPA (%), Inhibition of platelet aggregation in percent; MD, maintenance dose; LD, loading dose; o.d., once daily (every 24 h); b.i.d., twice daily (every 12 h).

Scenario	Treatment	Number of MD	Max. IPA (%)	IPA > 80% per Dosing Interval (h)	Time (%) with IPA > 80% per Dosing Interval
*Scenarios without loading dose of ticagrelor/prasugrel*					
1A	16 mg selatogrel (0 h) 600 mg clopidogrel (4 h) 90 mg ticagrelor b.i.d.	1	86.4	7.5/12.0	62.5
		2	92.8	11.7/12.0	97.5
		3	94.9	12.0/12.0	100.0
		4	95.7	12.0/12.0	100.0
		5	96.0	12.0/12.0	100.0
1B	16 mg selatogrel (0 h) 600 mg clopidogrel (4 h) 10 mg prasugrel o.d.	1	80.8	1.7/24.0	7.1
		2	97.8	23.4/24.0	97.5
		3	99.6	24.0/24.0	100.0
2A	16 mg selatogrel (0 h) 600 mg clopidogrel (12 h) 90 mg ticagrelor b.i.d.	1	89.6	10.6/12.0	88.3
		2	95.3	12.0/12.0	100.0
		3	96.7	12.0/12.0	100.0
		4	97.1	12.0/12.0	100.0
		5	92.7	12.0/12.0	100.0
2B	16 mg selatogrel (0 h) 600 mg clopidogrel (12 h) 10 mg prasugrel o.d.	1	88.7	18.0/24.0	75.0
		2	99.1	23.7/24.0	98.8
		3	91.9	24.0/24.0	100.0
*Scenarios with loading dose of ticagrelor/prasugrel*					
3A	16 mg selatogrel (0 h) 600 mg clopidogrel (4 h) 180 mg ticagrelor (16 h) 90 mg ticagrelor b.i.d.	0 (LD only)	96.2	11.3/12.0	94.2
		1	96.1	12.0/12.0	100.0
		2	96.1	12.0/12.0	100.0
		3	96.2	12.0/12.0	100.0
3B	16 mg selatogrel (0 h) 600 mg clopidogrel (4 h) 60 mg prasugrel (16 h) 10 mg prasugrel o.d.	0 (LD only)	100.0	23.6/24.0	98.3
		1	99.9	24.0/24.0	100.0
		2	99.8	24.0/24.0	100.0
4A	16 mg selatogrel (0 h) 600 mg clopidogrel (12 h) 180 mg ticagrelor (24 h) 90 mg ticagrelor b.i.d.	0 (LD only)	97.3	11.4/12.0	95.0
		1	97.5	12.0/12.0	100.0
		2	97.5	12.0/12.0	100.0
		3	97.4	12.0/12.0	100.0
		4	93.4	12.0/12.0	100.0
4B	16 mg selatogrel (0 h) 600 mg clopidogrel (12 h) 60 mg prasugrel (24 h) 10 mg prasugrel o.d.	0 (LD only)	100.0	23.6/24.0	98.3
		1	99.9	24.0/24.0	100.0
		2	94.8	24.0/24.0	100.0
*Scenarios with clopidogrel only*					
5	16 mg selatogrel (0 h) 600 mg clopidogrel (4 h) 75 mg clopidogrel o.d.	1	50.7	0.0/24.0	0.0
		2	19.0	0.0/24.0	0.0
		3	23.5	0.0/24.0	0.0
6	16 mg selatogrel (0 h) 600 mg clopidogrel (12 h) 75 mg clopidogrel o.d.	1	37.4	0.0/24.0	0.0
		2	28.8	0.0/24.0	0.0
		3	26.5	0.0/24.0	0.0

## Data Availability

Not applicable.
